# Understanding the contextual and causal factors shaping the work of receptionists in general practice: a realist review protocol

**DOI:** 10.1136/bmjopen-2025-110991

**Published:** 2025-12-23

**Authors:** Eleanor Hoverd, Megan E L Brown, Bryan Burford, Ko-Le Chen, Goran Erfani, Sameena Hassan, Anthony Montgomery, Matthew Lievesley, John Norton, Emily C Owen-Boukra, Tim Rapley, Nia Roberts, Madiha Sajid, Sarah Sowden, Alison Steven, Gillian Vance, Sophie Park

**Affiliations:** 1 Nuffield Department of Primary Care Health Sciences, University of Oxford, Oxford, UK; 2 School of Medicine, Newcastle University, Newcastle upon Tyne, UK; 3 Northumbria School of Arts and Creative Industries, Northumbria University, Newcastle upon Tyne, UK; 4 Deep End Network Northeast and North Cumbria ICS, Gateshead, UK; 5 Psychology, Northumbria University, Newcastle upon Tyne, UK; 6 Public Co-investigator, Southampton, UK; 7 Social Work, Education and Community Wellbeing, University of Northumbria at Newcastle, Newcastle upon Tyne, UK; 8 Bodleian Health Care Libraries, University of Oxford, Oxford, UK; 9 Public Co-investigator, London, UK; 10 Institute Health and Society, Newcastle University, Newcastle upon Tyne, UK; 11 School of Health and Life Sciences, Northumbria University, Newcastle upon Tyne, UK; 12 Newcastle University, Newcastle upon Tyne, UK; 13 University of Oxford, Oxford, UK

**Keywords:** General Practice, Primary Health Care, Health Workforce, Review

## Abstract

**Abstract:**

**Background:**

The work of receptionists in general practice is evolving rapidly and becoming more complex due to a number of changes within primary and community care services, such as increased digitalisation. In under-served areas, these changes have been further complicated by under-resourcing and workforce challenges around staff recruitment and retention. The National Health Service (NHS) 10-year health plan is set to accelerate further significant changes. There is limited understanding about how and why these changes and workforce challenges are impacting and will impact the future work of receptionists in general practice in under-served areas.

**Methods and analysis:**

This realist review will build on an existing programme theory related to general practitioner workforce sustainability. The review will examine what works, for whom, how and under what circumstances for receptionist work in general practice, in under-served areas. For example, how influences such as the expectations of patients (in under-served communities), poor staffing or limited career progression. Key stakeholders, including public contributors and individuals from general practice settings, will inform the realist review.

The review will be conducted using existing secondary and grey literature sources. The search strategy comprises five electronic databases: Medline, Embase, PsycINFO, CINAHL and Web of Science Core Collection (SCIE, SSCI, AHCI) with a date limit of 2015 applied to the search. The review will follow Pawson’s five steps: (1) shaping the scope of the review; (2) searching for evidence; (3) document selection and appraisal; (4) data extraction and (5) data synthesis. The findings will be reported in accordance with the Realist and Meta-narrative Evidence Synthesis Evolving Standards.

**Ethics and dissemination:**

Ethical approval is not needed for secondary analysis. The findings of this review will contribute to ongoing work as part of our ‘Workforce Voices’ programme of research. They will be disseminated to policymakers, commissioners, providers of health and social care and primary care and community healthcare teams through peer-reviewed publications, members of the public, conference presentations, social media and recommendations.

**STRENGTHS AND LIMITATIONS OF THIS STUDY**

- We will co-produce this realist review with patients and members of the public, as well as a stakeholder group of individuals from general practice that includes receptionists, increasing the relevance and rigour of the theory developed.
- A diverse range of evidence will be sought, including secondary and grey literature sources to capture the complexity of the topic, providing the necessary depth and breadth for identifying causal insights and refining programme theory.
- Our review may be limited by the quality and relevance of existing literature in this field.

## Introduction

 General practice, both in the UK and globally, is experiencing rapid transformation in service design and delivery, with increasing focus on accessibility, efficiency and sustainability.^1^,^5^ General practice sits within an increasingly complex landscape at the interface with primary care, actively collaborating with community-based services, third sector organisations and secondary care providers. Patients and policymakers expect faster access to general practitioner (GP) appointments, reflecting changing healthcare priorities.^3^

The 10 Year Health Plan for England (2025) is one of a number of key government policies that has developed the ongoing transformation of services in the National Health Service (NHS) focusing on three pivotal shifts.^1^,^6^ It aims to move healthcare closer to home, integrating more digital services to improve efficiency and patient care, focusing on preventative healthcare.^1 5^ Together with the rapid evolution of artificial intelligence (AI), this shift towards more digital service provision will continue to significantly impact on general practice mainly around how patients interact with general practice, with the government’s ambition for the NHS to be the most accessible health system in the world.^1^,^8^

Within the plan, there is a commitment to investing more in out-of-hospital care by 2029.^1^ Funding has historically been disproportionately allocated to secondary care services, with primary care attracting approximately 8.4% of the total NHS budget in 2023–2024 while delivering approximately 90% of NHS healthcare.^7^,^9^

The UK government announced a new budget for the Department of Health and Social Care in June 2025, seeking to address various areas of inequity such as housing, education and child poverty, social care and regional disparities.^10^ It outlines a commitment to increase funding, primary care workforce expansion, develop enhanced services and improve digital integration within the NHS.^10^ Despite decades of government policies to expand and invest in primary and community care services, there remains increasing demands on general practice, under-funding and a need for more efficient and equitable service delivery.^1^,^10^ The ‘Independent investigation of the NHS in England’ by Darzi provided an assessment of the current state of the NHS indicating a health system under significant strain which directly impacts the primary care workforce.^11^ Staff shortages across many roles have led to increasing workloads and higher levels of stress and burnout.^12 13^ Workforce shortages are further complicated by the multifaceted challenges that include financial constraints, varying levels of digital maturity and the expansion of services and new roles - all of which are aimed at modernising primary care and providing an efficient, effective and equitable service.^15^,^10 14^

### The work of general practice receptionists

General practice in the UK historically provides universal, comprehensive healthcare for all who seek it and for any problem, within their local population. Receptionist roles are at the interface between the public and healthcare services and are often the first point of contact in general practice.^15 16^ Their work involves multiple responsibilities, guiding patients through complex systems while ensuring efficient operation of general practice teams, directly affecting workflow management.^17^,^22^ Some patients may present with clearly defined requests, while others will seek help for undifferentiated and/or multiple needs.^23^ With expectations of healthcare changing, receptionist roles navigate boundaries between healthcare ‘needs’ and ‘wants’. This is particularly salient in the context of current UK policy set out in the 10 Year Health Plan for England (2025).^1^

Responsibilities that accompany receptionist roles are expanding due to recent initiatives that aim to improve patient access, making it quicker and easier for patients to get appointments, reduce pressure on general practices and modernise service delivery.^315^,^24^ However, these changes mean that the work of the receptionist is becoming more complicated while impacting on their work experiences and the wider general practice workforce.^15^,^17^

Reports suggest an increasing incidence of aggression and hostility as receptionists are increasingly subject to aggression and hostility from patients, which has worsened since the COVID-19 pandemic.^18 19^ Negative media portrayals of GP appointment access have further heightened tensions between patients and general practice staff, contributing further to a hostile working environment.^25^ Many receptionists report low job satisfaction due to insufficient support from senior staff and lack of recognition.^17^ This issue is particularly pronounced in under-served areas, where practices face resource limitations and serve populations with restricted healthcare access.^26 27^

### Challenges facing under-served areas

Access to healthcare varies with geographical, social and socioeconomic context, and some areas are consequently ‘under-served’; although the term is problematic as it is considered context-dependent.^28^ Efforts to address health inequalities seek to provide better services for groups who are often excluded from services due to no fault of their own.^28^ Some under-served groups face multiple disadvantages such as the intersection of race and gender, known as intersectionality.^29 30^ Therefore, there are different ways in which someone may be under-served, and so the development of future interventions should account for these differences.^28^,^30^

Some localities, including remote, rural and coastal locations, and areas of high deprivation, face significant challenges of delivering care where health inequalities may contribute to and be compounded by workforce challenges such as shortages of staff in general practice, high patient demand and limited resources.^326^,^32^ In these under-served areas, there are fewer GPs per 10 000 patients compared with well-resourced areas.^12 13 28^ These challenges impact not only on clinical delivery but also place a significant burden on non-clinical staff, such as receptionists, who often experience increased workloads and stress, alongside low pay, leading to burnout and high turnover rates.^15^,^17^

### Adaptation of general practice receptionist roles

The evolving dynamic of an increasingly complex role, unclear role boundaries, increased workload and system changes within the NHS exacerbate issues of stress, burnout, retention and recruitment for receptionists.^17^ Widening of the general practice receptionist role, scope of work and the language used to describe a range of roles demonstrates the spectrum of non-clinical support roles that are expanding to cope with the increased pressures on general practice and digitalisation. Table 1 shows the multifaceted nature of non-clinical support roles, categorising the type of role, title, core functions and whether interaction with patients is indirect or direct. The work of receptionists may involve a range of these responsibilities, with the role of general practice assistants often being filled by experienced receptionists.^17^ This provides a framework to reflect on when considering what role expectations are and what receptionists actually do in their daily work lives. This is not an exhaustive list as it is recognised roles and role titles may vary among general practices.

**Table 1:** Types of non-clinical support roles in general practices

Type	Patient interaction	Role titles	Key areas of work
Frontline care coordinator	Direct	Receptionist, senior receptionist, reception manager	Booking appointments, answering phones, signposting patients, managing administrative queries
Support access	Direct	Care navigator/care coordinator	Guiding patients to appropriate healthcare professionals
Workflow, practice support	Indirect	Administrator, medical secretary	Mostly work in the ‘back-office’, summarising new patient notes, scanning of documents, processing referral requests, managing referrals, handling incoming and outgoing documents
	Direct	GP assistants	Provide some clinical support through preparing patients for examination, assisting with minor procedures, updating medical records, typing referral letters, managing clinical correspondence

GP, general practitioner.

Without adequate support and investment, the evolution of the work associated with the role of receptionists and what the real, lived experience of doing the job is, may have implications for the sustainability of this key group within the general practice workforce. If the scope of work exceeds and outpaces the initial scope of job roles, problems may arise.^17^,^1922^

### Workforce sustainability

This review is being conducted as part of a programme of work undertaken by Workforce Voices, an National Institute for Health and Care Research (NIHR)-funded Workforce Research Partnership (NIHR160772). This partnership will be conducting a number of studies to support workforce sustainability in under-served areas (https://worforcevoices.org).

The Partnership team brings together academics from five Higher Education Institutes across the North East, Oxford, York and Birmingham, England, with expertise in the healthcare workforce, public health, primary care, clinical education, social and behavioural science, human resource management, organisational psychology and health economics.

Workforce sustainability requires attention to a range of factors including financial and environmental, human and capacity building.^31^ While there are varying definitions of workforce sustainability, many include efforts to support stability; adaptability to future demands; and work approaches which minimise environmental impact that includes three pillars of sustainability: social, environmental and economic.^33^ We will collaborate with patients and members of the public and stakeholders to review existing definitions of workforce sustainability and reach a consensus on a definition that best aligns with our shared objectives.

### Why this review is important

Evidence suggests that NHS receptionist turnover is reaching unprecedented levels and so a realist review offers a crucial opportunity to understand not just what is happening, but why, how and under what circumstances, ensuring that interventions are tailored to the realities of working on the frontline.^16 17^ It is pertinent to understand how changes to processes such as the digitalisation of the NHS are affecting the work, experience and outcomes of general practice receptionists in relation to workforce sustainability and equitable patient care. It is the ambition of the 10 Year Health Plan for England to develop an NHS workforce ‘fit for the future’.^1^

A realist review will provide causal explanations of the factors shaping general practice receptionists’ work and the wider general practice workforce that have not been explored in prior systematic reviews.^17 18^ A previous systematic review identified gaps related to a lack of effective training programmes, role definition, workload challenges, public perceptions of receptionists and the impact of digitalisation.^17^ It did not explore, or articulate, the underlying mechanisms driving these issues around gaps in knowledge, learning, training, support and the impact on service provision. Given the urgent need within the NHS to address workforce challenges in general practice—where equitable access and continuity of care are paramount, particularly in under-served communities—this review will shed light on the conditions influencing receptionist recruitment, retention and how these impact on wider workforce sustainability and equitable access to patient care.^5 31^

Paradoxical tensions of increasing digitalisation, which can increase workload, as well as the fear of AI potentially threatening job existence, are a serious concern, yet we do not understand fully the role and/or implications of delegation to more algorithmic approaches.^34^ Therefore, this review is imperative to understand the key tenets of receptionist work. This examination is critical now to ensure that assumptions about the ability of AI to replace receptionist work are not made at pace, without understanding the complex terrain and nature of these roles.

Early scoping conversations with a range of staff in under-served areas highlighted the challenges associated with the work receptionists in general practice are carrying out, as a key workforce sustainability priority. By addressing gaps from a previous systematic review on training, role definition, workload, burnout, public attitudes and digitalisation, we aim to provide insights for service design and workforce planning, helping policymakers and primary care leaders improve workforce sustainability and equitable patient access, care and a positive experience in under-served areas.^26 27 29^

## Methodology

### Realist review

The role of the modern general practice receptionist is multifaceted, reflecting the complexity of the system which it supports.^15 6 8 11 15^,^17 21 22^ In realist terms, a system is a complex and open set of relationships, structures and processes influenced by sociopolitical, cultural and historical factors.^35^ Interventions are implemented in systems with outcomes (intended and unintended) that emerge dependent on how context shapes mechanisms.^35^ Through taking a realist approach, we can make sense of this complexity by understanding how systems constrain and shape interactions, examining the varying contexts that exist and how people working with general practice interact with existing interventions, or programmes such as new digital services.

**Figure 1:**
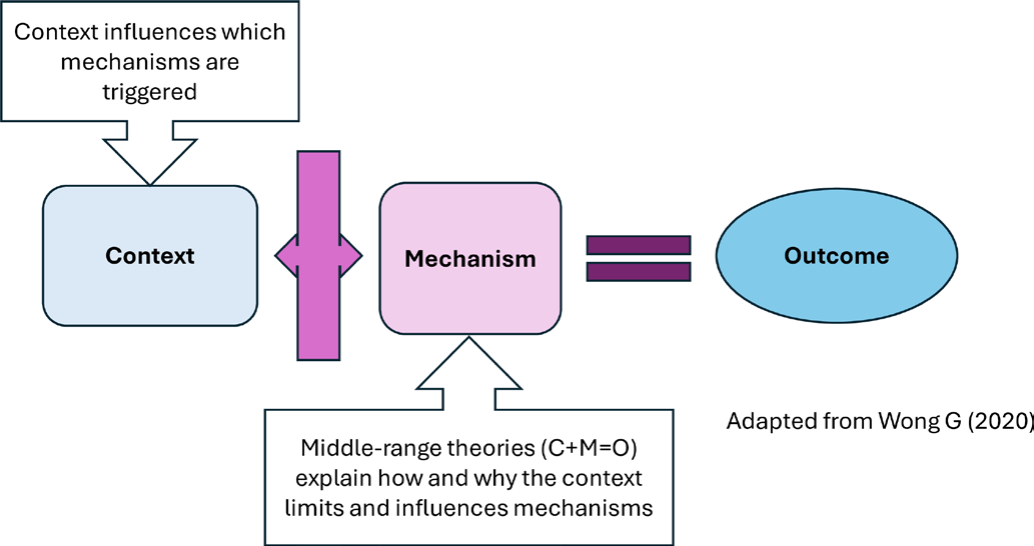
Illustration of middle-range theory expressed as CMOC. CMO, contexts, mechanisms and outcomes; CMOCs, CMO configurations.

Realist reviews seek to develop theories using an explanatory approach.^36^ They use a broad range of evidence to unpack the relationships between contexts (C), mechanisms (M) and outcomes (O), known as CMO configurations (CMOCs).^36 37^ These CMOCs represent and illustrate theoretical explanations of causation expressed as middle-range theories (MRTs).^35 38^ The main goal of a realist review is to uncover MRTs. Contexts shape the way mechanisms and outcomes are produced as shown in figure 1.^35 36^

A theoretical explanation of how and why outcomes vary in relation to the work of receptionists across different contexts is lacking in the evidence base.^15^,^22^ Overall, realist reviews can help to explain the deeper, often hidden, causal factors that go beyond understanding if something is effective, to understanding why a particular intervention functions and flourishes in one context and not another.^39 40^ There is a gap in understanding the system changes and how they shape the work of the receptionist, workforce sustainability, equitable patient access, care and a positive patient experience in under-served areas. This realist review will advance knowledge through examining the mechanisms and contextual factors that shape the work of receptionists, how and when the role of the receptionist works well and what the outcomes are in relation to workforce sustainability and equitable patient access, care and positive experience in under-served areas. The review will produce causal explanations as described by Pawson in the form of context-mechanism-outcome configurations that create a ‘medium of policy learning’ which is under-reported in the literature. This review will also create a practical blueprint for the implementation of interventions. This will be applied to the ‘Workforce Voices’ programme of work and reported on in future publications as well as any findings that have led to system change.

### Building on findings from a previous realist review on GP workforce sustainability

A realist review usually begins with an initial programme theory (IPT). A programme theory (PT) explains how, why, for whom and under what circumstances an intervention works.^37^ An IPT can be developed in a range of ways. Often, a review happens in isolation and will relate only to the specific piece of work. This review aims to extend and build on a previous realist review that focused on examining how general practices and healthcare systems support the work of the GP workforce in the UK.^31^ It identified three key areas in its resultant PT, as shown in table 2.^31^ The three key areas were underpinned by 18 CMOCs.

**Table 2:** Key areas of GP workforce sustainability PT

Key area	Explanation
Meaningful aspects of clinical practice	GPs require work that is purposeful, significant and aligns with their core values. There are different contributing and mitigating factors that impact on whether work is meaningful and engaging. For example, time spent on meaningful aspects of practice, for example, doctor-patient interactions resulting in better engagement with their work.
Relationships across individuals, organisations and communities	Knowledge accumulation that occurs through long-term patient-GP relationships facilitates more appropriate patient care. Familiarity with local areas and communities supports knowledge accumulation and in turn benefits patients and communities. Working with colleagues and patients nurtures meaningful practice and improved experiences of connection and engagement.
Learning and development	Learning systems that promote regular connections with colleagues, informal interaction and peer support contribute to GPs feeling connected and better able to cope. Informal engagement and peer support create a sense of community that helps GPs to thrive and has several other benefits like increasing patient safety.

GP, general practitioner; PT, programme theory.

While the review of GP workforce sustainability highlighted the broader context and mechanisms influencing GP workforce sustainability, this realist review will provide a specific focus on the changing system of primary care affecting general practice receptionists.^31^ The translation of these three key areas to non-clinical staff is a significant step, though sensical when adapted to the work of receptionists. This review will examine how these key areas align with, extend thinking, refute thinking or bring new ideas in relation to the work of receptionists in general practice, in under-served areas.

### Generalisability and transferability of findings

Emmel *et al* point out that ‘social programmes do not work always and everywhere’.^41^ However, realist reviews can generate theoretical generalisability in that the search for evidence continues until new evidence does not extend thinking further, or contradicts what has already been found.^41^ Findings from realist reviews can be transferable to different settings, supporting a contextual approach to decision-making.^39^ This is often achieved through generating theories which are applicable to multiple and/or different settings, which is how they are generalisable.^41^ While CMOCs and programme theories can need adaptation to maximise relevance to particular contexts, there can be commonalities in the causal forces across settings.^42^

Through co-production, we will listen to content experts and inductively connect grassroots knowledge with insights from the previous GP workforce sustainability review and new findings from this review.^31^ Retroductive thinking that occurs in having these discussions enables the identification of middle-range mechanisms that may have a wider relevance.^40^ MRTs explain how and why the context limits and influences mechanisms, and it is argued by Mukumbang *et al* that middle-range mechanisms can help to identify and achieve generalisability.^42^ Through contextualising research findings in a realist review, an assessment of their transferability can be made when there is a clear articulation of contextual factors.^42^

This protocol recognises that applying synthesised research goes beyond academic contexts to make sure the findings are actionable and reflect real-world complexities.^38 40^ The development of a PT enables informed judgments about how/if/when findings might be applicable elsewhere. This recognises the socially situated nature of interactions and intervention implementation.

Building on a previous review and PT through taking this approach will strengthen the relevance and generalisability of findings. While aiming to develop a PT around the work of receptionists in general practice, the GP workforce sustainability PT provides a rigorous, empirically grounded starting point to explore whether mechanisms are relevant for receptionists and whether context needs to be redefined for the work of receptionist roles. We will also explore how resulting outcomes differ. A co-produced realist review provides a broader foundation for deeper insights and more adaptable implementation strategies, through engagement with a range of knowledge (eg, experiential knowledge, grey literature and empirical research). This cumulative process enables us to build on insights gained from analysing and synthesising a number of studies and the findings of the GP workforce sustainability review. By identifying new ways of understanding and interpreting these findings, we can apply them meaningfully to shape our future ‘Workforce Voices’ co-design work across the wider workforce sustainability programme workstreams. Formal theories of change and implementation will provide conceptual scaffolding to guide the realist reviews, co-design and implementation work.^43 44^

### Realist review methodology within the context of the ‘workforce voices’ partnership

Insights from this review will inform the broader programme logic across the workforce partnership.

### Methods and analysis

#### Aim

This realist review will explore empirical and grey literature on the work of receptionists in under-served areas of general practice to build on the existing PT concerning GP workforce sustainability.^31^ The aim will be met through answering the following research questions:

1. What are the opportunities and challenges of being a receptionist in general practice in under-served areas?
2. What are the contextual factors at each level of the health and social care system which impact on receptionists’ work, the challenges and opportunities that they are presented with, their interactions with patients and workforce sustainability?
3. What interventions exist, or are required, to assist reception staff in adapting their work over time?
4. What are the outcomes of changes within the system to receptionists’ work on equitable and effective delivery of patient care, reception staff experiences and workforce sustainability?

### Study design

**Figure 2:**
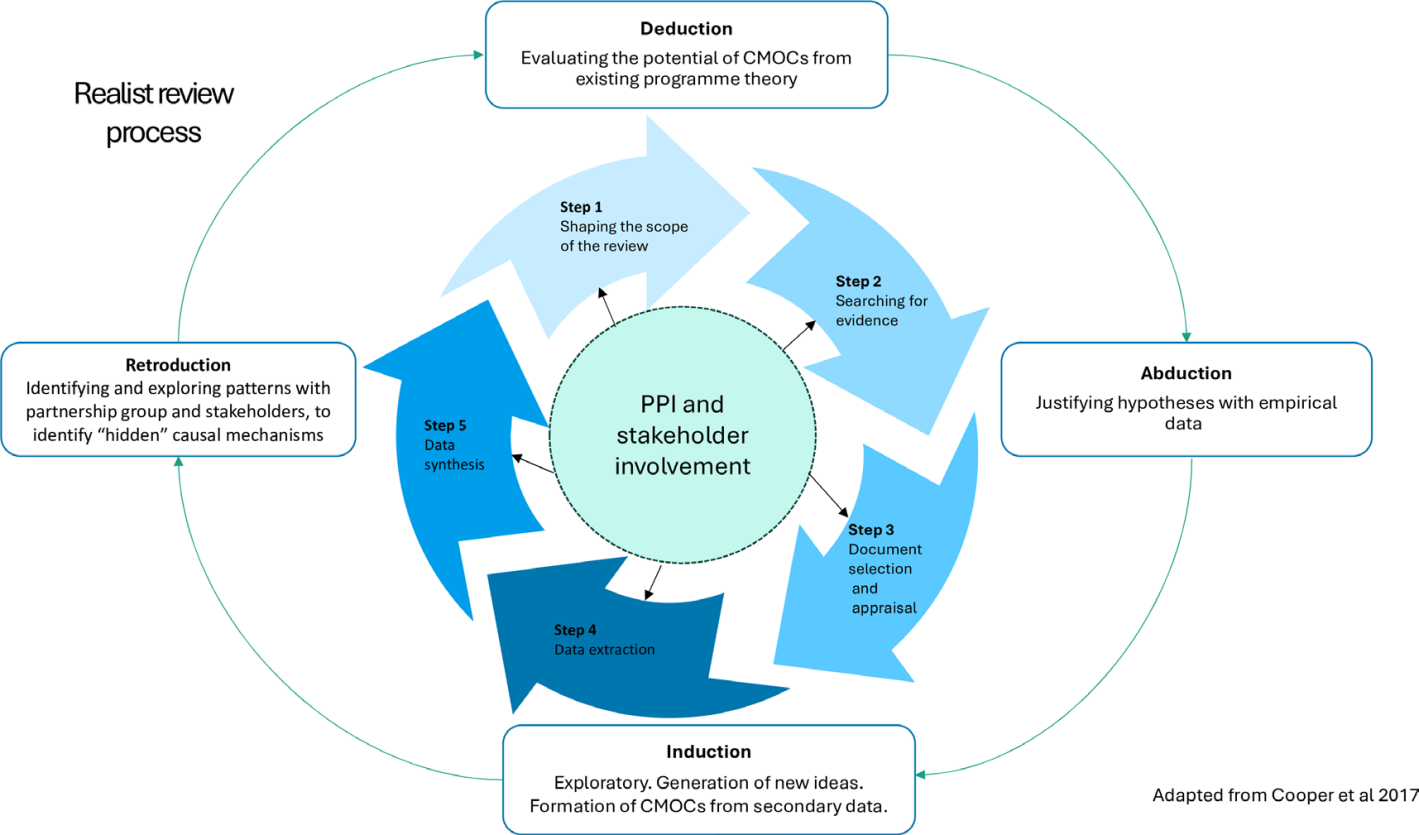
Realist review process. CMO, contexts, mechanisms and outcomes; CMOCs, CMO configurations; PPI, patient and public involvement.

The study design will be guided by Pawson et al’s five steps and in accordance with Realist and Meta-narrative Evidence Synthesis Evolving Standards for quality and reporting.^37^ Figure 2 shows a diagram of the review process adapted from Cooper *et al*.^45^

### Stakeholder and patient and public involvement (PPI)

Scoping conversations served to elicit PPI and stakeholder input refining the focus of the review. We presented the GP workforce sustainability review findings to a range of groups in general practice from six regions around England, captured their responses and used these to inform plans and priorities for this realist review. This helped to ensure the review questions are targeted and responsive to the needs of patients and the general practice workforce.

We will co-produce this review with stakeholders, who will remain actively involved throughout the review. We will apply the principles of co-production: sharing of power, including all perspectives and skills, respecting and valuing the knowledge of stakeholders, ensuring reciprocity and building and maintaining relationships.^45 46^ Stakeholders include a range of content matter experts working within general practice and also patients and members of the public, with an interest in shaping the research and may be affected by its outcomes.^41^ A Community Involvement Engagement (CIE) lead (MELB) for the workforce research partnership will oversee PPI and involvement from stakeholders.

#### Recruitment of stakeholders (content experts)

Two members of the public, two receptionists working in general practices, one practice manager and one individual at the commissioning level of the healthcare system will form the content expert group for this realist review.^41^ Public contributors were invited via a CIE group that has been established for the ‘Workforce Voices’ programme of work. This CIE group consists of both patients, members of the public and individuals who work in the NHS. Patients and members of the public will help to ensure the review remains relevant to the needs of patients in under-served areas of general practice. In partnership with the content expert group, EH will establish key project milestones where active involvement will be sought. The group will be consulted via virtual meetings throughout the review that will last no longer than 1.5 hours to prevent digital fatigue.^47^ Content experts will be renumerated as per NIHR payment and guidance advice.^48^ All meetings will be inclusive with intentional and accessible planning, active facilitation and reflection incorporated. A terms of reference agreed with the group will be developed with flexible approaches taken to meet the diverse needs of the group.

### Steps in the review process

The review will be conducted according to the following five steps: (1) shaping the scope of the review, (2) searching for the evidence, (3) document selection and appraisal based on relevance and rigour, (4) data extraction and organisation of the evidence and (5) data synthesis to refine the PT.^35^

### Step 1: shaping the scope of the review

A realist review on GP workforce sustainability (NIHR project number 593, funding round FR4) developed by several of the co-authors will provide a PT to build on, described by Pawson as ‘reusable conceptual platforms’ to aid further discovery (see online supplemental file 1).^31 49^ We will draw on this PT as well as concepts critical to the work of receptionists in general practice through engaging in discussions with public contributors, stakeholders and the partnership group.

### Step 2: searching for evidence

A search of secondary data sources will be conducted, including published quantitative, qualitative and mixed-methods empirical studies, in addition to grey literature that has not been traditionally published such as blogs, conference abstracts and reports by government.^41^

A database search strategy was developed in May 2025 by NR and EH through drawing on knowledge of searches used in the GP workforce sustainability realist review.^31^ A pilot search of the evidence to assess feasibility of the review was conducted on Medline, yielding an initial 66 sources. Subsequent scoping of these sources indicated that they were relevant to the review’s focus. A date limit of 2015 will be applied to the search. This reflects the changes in policy over the last 10 years that have contributed to the shifts and changes in receptionists’ work in general practice since 2015.^1^,^10^

We anticipate further adjustments to be made to the search strategy, though current search strategies for all databases are included in online supplemental file 2. While international insights can be valuable, our primary focus will remain on specific contexts impacting the work of receptionists in GP practices in the UK. Receptionist roles vary internationally; in the USA, receptionists manage insurance processes and coordinate specialist referrals.^50^ We will consider integrating relevant international literature, or from contexts where a publicly funded healthcare system is in place free at the point of need, closely aligned to arrangements in the UK, in iterative searches if it becomes pertinent to our review. Emerging evidence will be discussed with stakeholders and the core ‘Workforce Voices’ group (research team) to agree by consensus as to whether an iterative search should widen the review to international evidence. This may be limited by time and resource, but we will be guided by asking whether doing so may help to answer the research questions and whether doing so will add to PT development. A data extraction form will be developed, in collaboration with public contributors and key stakeholders and informed by a realist review on GP workforce sustainability. Evidence will be scored as high, moderate or low based on relevance and usefulness.^49^ The data extraction form will include full reference, source type, country of publication, quality and the group being researched, although this will be developed iteratively to meet the needs of the review and amendments reported on in a subsequent realist review publication.

#### Data sources and search strategy

**Figure 3:**
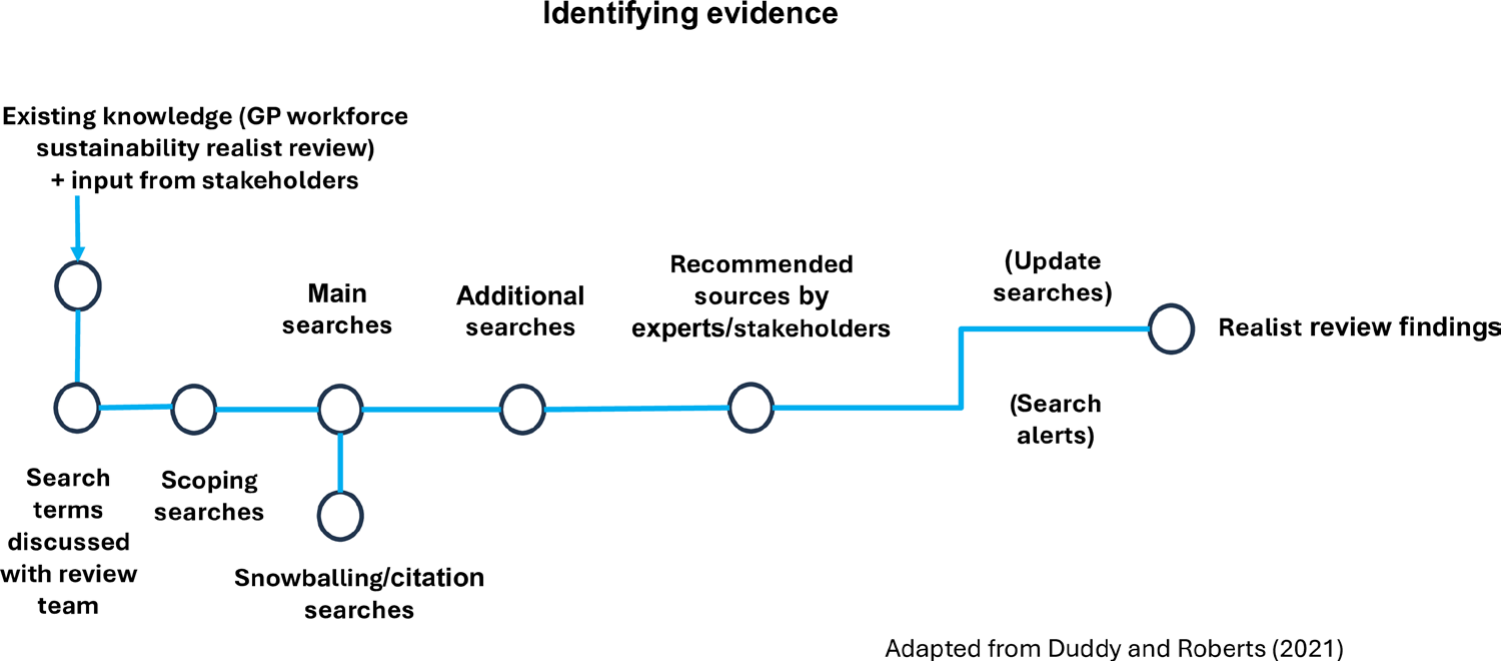
Identifying evidence. GP, general practitioner.

An initial search will be adapted from the searches developed in the GP workforce sustainability project.^31^ Search terms will be developed in discussion with the review team. Figure 3, adapted by Duddy and Roberts, shows how evidence will be identified.^51^

An example of the search strategy for Medline can be found in online supplemental file 2. The search strategy was based on a three-concept search: receptionists, general practice and United Kingdom. Endnote referencing software will be used for storing sources.^52^ The strategy will support a search for the evidence on the following databases: Medline, Embase, PsycINFO, CINAHL and Web of Science Core Collection (SCIE, SSCI, AHCI) including academic and grey literature from health-focussed and broader disciplines. Recommendations from public contributors, the partnership group and stakeholders will also inform the grey literature search.

Once the initial search is completed, public contributors, the partnership group and stakeholders will decide if further literature searching is required.

#### Screening process

Inclusion criteria will be determined collaboratively with PPI and stakeholders and focus on three key areas: (1) work experiences, (2) learning, training and support and (3) impact on service provision/implementation . We will select data to maximise included documents’ contributions to our evolving programme theories. We will follow a realist logic of analysis to inform data extraction from a final set of included documents, and to formulate CMO configurations and contribute to the developing PT.^35^,^39^ Implications of CMOs for workforce sustainability and equitable patient outcomes will be explicitly considered.

EH will undertake screening based on title and abstract, and a second reviewer, ECO-B, will screen a random sample of 10% of the identified citations. Reviewers will discuss disagreements that cannot be reconciled, with the partnership and stakeholder groups. Full screening of each document will be carried out at second level screening. EPPI-Reviewer software will be used for screening, managing and analysing data.^53^ Papers excluded by one reviewer but included by the other will be discussed with public contributors, the partnership group and stakeholder group, including those where either one or two reviewers are uncertain.

#### Iterative searching

The iterative nature of realist reviews may require searching for additional data to support PT development.^41^ For additional searches, further inclusion/exclusion criteria will be developed and subsequent searches tested and refined with the information specialist NR.

### Step 3: selection and appraisal

Following initial screening in step 2, sources will be selected according to rigour and relevance.^54^ Relevant sources are those that are felt to be valuable for building theory with rigour relating to the trustworthiness and credibility of the methods used to produce data.^54^ However, Pawson advises that even sources of poor methodological quality can offer important insights about causal factors and should be included if that is the case.^49 54^ Therefore, we will consider evidence that appears to be of poorer methodological quality if felt to be relevant to the development of PT.^35 37 41^ Quality-assessment checklist criteria will not be used as is standard in realist reviews.^35^ However, the relevance and rigour will be assessed as outlined in step 5.^35^

### Step 4: extracting and organising data

Data will be extracted by EH with reviewer 2 (ECO-B) completing a 10% subsample. Disagreements will be dealt with as discussed in step 2. EPPI-Reviewer will be used to assist with data coding and organisation, with Excel used, if required, to support organisation of data.^51^ Extracted data will focus on narratives that support PT development. Codes will be developed inductively from the data and deductively from the existing PT.^31 54^ Patterns will be identified in the data through grouping the codes together and prioritising coding for causal insight in regard to the contextual factors and mechanisms which impact on receptionists’ work, their interactions and experiences, and workforce sustainability including recruitment and retention. There may be instances where only a partial CMOC can be built. Partial CMOCs will still be included as evidence may be available within another source to complete them, or we may be able to hypothesise a complete CMOC and search for further evidence to confirm, refute or refine it.^41^ Where possible, given any potential limitations of the data contained within the included documents, we will specify the PT to take into account the role of under-served areas in our CMOCs. This will support identifying patterns in the data and configuring data into CMOCs. CMOCs will be mapped against the existing PT (GP workforce sustainability) that align with existing theories, refute existing theories, introduce new CMOCs or extend thinking (online supplemental file 1).^31^

### Step 5: analysis and synthesis

Realist logic of analysis will be applied to included data in the review.^3537^,^39^ This requires building on and refining the IPT through drawing on the coding carried out in EPPI-Reviewer to build CMOCs.^52^ CMOCs will be compiled through synthesising data across sources. The relevance and rigour of included sources are essential for assessing how well a piece of evidence informs the development of PT and how credible and trustworthy it is.^29 39 55^ A set of questions adapted from Papoutsi *et al*^55^ combined with the criteria purported by Duddy and Wong will be used to guide this process as shown in table 3.^55 56^

**Table 3:** Assessing relevance and rigour

Relevance and consilience	Identify as much relevant (diverse) evidence as possible Do the contents of a section of text within an included document refer to data that might be relevant to programme theory development?
Judgements about trustworthiness and rigour	Are the data sufficiently trustworthy to warrant making changes to the programme theory
Interpretation of meaning	If a section of text is relevant and trustworthy enough, do the contents provide data that may be interpreted as being context, mechanism or outcome?
Interpretations and judgements of CMOCs	What is the CMOC (partial or complete) for the data? Is there data to inform CMOCs contained within this document or other included documents? If so, which other documents? How does this CMOC relate to CMOCs that have already been developed?
Interpretations and judgements about programme theory and simplicity	How does this (full or partial) CMOC relate to the programme theory? Within this same document, is there data to inform how the CMOC relates to the programme theory? If not, is there data in other documents? Which ones? In light of this CMOC and any supporting data, does the programme theory need to be changed? Aim for conceptual clarity, avoiding complex explanations
Analogy	Does programme theory fit with what is already known in the field? Does it extend or refine existing knowledge?

CMO, contexts, mechanisms and outcomes; CMOC, CMO configuration.

Data analysis and synthesis will be led by EH, with reflections and discussions among the partnership group and stakeholder group to aid interpretation of CMOCs through retroductive thinking. Retroductive thinking is a form of reasoning that supports the identification of ‘hidden’ causal mechanisms that occur behind observed patterns.^57^ It is used alongside abductive thinking that allows educated guesses, or ‘hunches’ to be made about the likely explanation for what is being observed in relation to outcomes.^57 58^ Involving stakeholders and the partnership group in this theorising will make sure that the PT reflects their experiences and will validate interpretation of CMOCs.

### Strengths and limitations

A key strength of the review is working collaboratively with stakeholders (including public contributors) and the partnership group as they consist of practitioners working within the NHS, holding extensive expertise across health services and workforce research. This will contribute to the rigour and relevance of the review through ensuring collective insight and expertise is incorporated. The review may be relevant to wider primary care and community services based in under-served areas but also in well-resourced areas. Our review may be limited by the quality and relevance of existing literature in this field and the extent to which under-served communities are consistently included, referenced and indexed. The review may also be of relevance internationally as similar challenges exist around the globe.^59^ Included sources will represent a broad range of grey literature, policy reports and conference papers to create breadth and depth to the review. Through working with stakeholders, we will prioritise any aspects of PT that we are unable to address during this review and consider them as future research projects.

### Ethics and dissemination

Ethical approval is not required for a realist review of secondary sources of evidence. The realist review is registered with PROSPERO (1085929). Throughout the review, ethical considerations will be reflected on, documented and reported in the realist review findings, with consideration of the power dynamics that may exist between the stakeholder group and partnership group. Findings will include a PT that will support the development of co-designed, rapid interventions and implementation of them as part of the ‘Workforce Voices’ programme of work. Dissemination will be planned with the content expert group, with further suggestions by the partnership research advisory group and CIE panels made as the review progresses. These will be reported on in a subsequent publication.

## Supplementary material

10.1136/bmjopen-2025-110991 online supplemental file 1

10.1136/bmjopen-2025-110991 online supplemental file 2
