# Supplementary material for: Understanding the contextual and causal factors shaping the work of receptionists in general practice: a realist review protocol
Source: BMJ Open. 2025 Dec 23;15(12):e110991. doi: 10.1136/bmjopen-2025-110991 (PMC12730864; doi:10.1136/bmjopen-2025-110991)
Supplement: online supplemental file 1 [file bmjopen-15-12-s001.docx]

**Supplemental file 1. Refined programme theory.**

| **Facilitators** | |
| --- | --- |
| **Meaningful work and engagement** | - 1. Congruence between GPs’ core values and the nature of their work   2. Opportunities for reciprocal care and mutual acts of compassion   3. Balance work demands and available resources (e.g. appropriate consultation time)   4. Support GP roles as advocates and enable GP/patient agency   5. Recognise and enable intellectual stimulation in GP work (e.g. agile and flexible expertise to personalise/contextualise care) |
| **Relationships across individuals, organisations, and communities** | - 1. Connection-rich contexts (direct interactions and connections within work activities)   2. Cultivate and use cumulative knowledge (regarding local people and place) to inform the organisation and delivery of care   3. Facilitate direct connections and cross-disciplinary learning opportunities between peers and organisations   4. Enable informal learning, engagement, and peer support |
| **Learning and development** | - 1. Climate of psychological safety that enables opportunities for care and on-going practice-based learning with patients and peers   2. Embed spaces for learning and exchanging cumulative knowledge into learning structures and systems   3. Promote enabling cultures and dynamic learning systems to facilitate the negotiation of risk, ambiguity, and uncertainty |
| **Barriers** | |
| - 1. Depersonalisation and commodification of GP work   2. Lack of recognition/planning to support potential disconnection and additional work inherent in remote consulting   3. Paradox of delegation to supervise and support allied HCPs | |
