# Supplementary material for: Understanding the contextual and causal factors shaping the work of receptionists in general practice: a realist review protocol
Source: BMJ Open. 2025 Dec 23;15(12):e110991. doi: 10.1136/bmjopen-2025-110991 (PMC12730864; doi:10.1136/bmjopen-2025-110991)
Supplement: online supplemental file 2 [file bmjopen-15-12-s002.docx]

Supplementary file 2

Search strategies

| **Medline** |
| --- |
| Medline (Ovid MEDLINE® Epub Ahead of Print, In-Process & Other Non-Indexed Citations, Ovid MEDLINE® Daily and Ovid MEDLINE®) 1946 to present  1 Medical Receptionists/ 162  2 (receptionist? or reception staff*).mp. 723  3 1 or 2 723  4 exp General Practice/ or General Practitioners/ or Physicians, Family/ 103562  5 general pract*.ti,ab,kf,jw. or bjgp.jw. 111870  6 4 or 5 165765  7 3 and 6 310  8 exp United Kingdom/ 405304  9 (national health service* or nhs*).ti,ab,in. 315518  10 (english not ((published or publication* or translat* or written or language* or speak* or literature or citation*) adj5 english)).ti,ab. 144335  11 (gb or "g.b." or britain* or (british* not "british columbia") or uk or "u.k." or united kingdom* or (england* not "new england") or northern ireland* or northern irish* or scotland* or scottish* or ((wales or "south wales") not "new south wales") or welsh*).ti,ab,jw,in. 2675800  12 (bangor or "bangor's" or cardiff or "cardiff's" or newport or "newport's " or st asaph or "st asaph's" or st davids or swansea or "swansea's").ti,ab,in. 79263  13 (aberdeen or "aberdeen's" or dundee or "dundee's" or edinburgh or "edinburgh's" or glasgow or "glasgow's" or inverness or (perth not australia*) or ("perth's" not australia*) or stirling or "stirling's").ti,ab,in. 286698  14 (armagh or "armagh's" or belfast or "belfast's" or lisburn or "lisburn's" or londonderry or "londonderry's" or derry or "derry's" or newry or "newry's").ti,ab,in. 38534  15 (bath or "bath's" or ((Birmingham not alabama*) or ("birmingham's" not alabama*) or bradford or "bradford's" or brighton or "brighton's" or bristol or "bristol's" or carlisle* or "carlisle's" or (cambridge not (massachusetts* or boston* or harvard*)) or ("cambridge's" not (massachusetts* or boston* or harvard*)) or (canterbury not zealand*) or ("canterbury's" not zealand*) or chelmsford or "chelmsford's" or chester or "chester's" or chichester or "chichester's" or coventry or "coventry's" or derby or "derby's" or (durham not (carolina* or nc)) or ("durham's" not (carolina* or nc)) or ely or "ely's" or exeter or "exeter's" or gloucester or "gloucester's" or hereford or "hereford's" or hull or "hull's" or lancaster or "lancaster's" or leeds* or leicester or "leicester's" or (lincoln not nebraska*) or ("lincoln's" not nebraska*) or (liverpool not (new south wales* or nsw)) or ("liverpool's" not (new south wales* or nsw)) or ((london not (ontario* or ont or toronto*)) or ("london's" not (ontario* or ont or toronto*)) or manchester or "manchester's" or (newcastle not (new south wales* or nsw)) or ("newcastle's" not (new south wales* or nsw)) or norwich or "norwich's" or nottingham or "nottingham's" or oxford or "oxford's" or peterborough or "peterborough's" or plymouth or "plymouth's" or portsmouth or "portsmouth's" or preston or "preston's" or ripon or "ripon's" or salford or "salford's" or salisbury or "salisbury's" or sheffield or "sheffield's" or southampton or "southampton's" or st albans or stoke or "stoke's" or sunderland or "sunderland's" or truro or "truro's" or wakefield or "wakefield's" or wells or westminster or "westminster's" or winchester or "winchester's" or wolverhampton or "wolverhampton's" or (worcester not (massachusetts* or boston* or harvard*)) or ("worcester's" not (massachuse tts* or boston* or harvard*)) or (york not ("new york*" or ny or ontario* or ont or toronto*)) or ("york's" not ("new york*" or ny or ontario* or ont or toronto*))))).ti,ab,in. 1948554  16 8 or 9 or 10 or 11 or 12 or 13 or 14 or 15 3435792  17 (exp africa/ or exp americas/ or exp antarctic regions/ or exp arctic regions/ or exp asia/ or exp oceania/) not (exp great britain/ or europe/) 3581699  18 16 not 17 3217179  19 7 and 18 194  20 limit 19 to yr="2015 -Current" 66  21 practice staff.mp. 899  22 20 or 21 |
| **EMBASE** |
| Embase 1974 to present  1 Medical Receptionists.mp. or medical receptionist/ 98  2 reception staff.mp. 175  3 General Practice.mp. or general practice/ 108474  4 General Practitioners.mp. or general practitioner/ 152716  5 Physicians, Family.mp. or general practitioner/ 131275  6 1 or 2 267  7 3 or 4 or 5 234488  8 6 and 7 108  9 United Kingdom.mp. or United Kingdom/ 603529  10 national health service.mp. or national health service/ 87625  11 8 and 9 20  12 limit 11 to yr="2015 -Current" |
| **PsychINFO** |
| PsycINFO 1806 to present  1 Medical Receptionists.mp. 17  2 (recptionist? or reception staff*).mp. [mp=title, abstract, heading word, table of contents, key concepts, original title, tests & measures, mesh word] 22  3 exp General Practice/ or General Practitioners/ or Physicians, Family/ 6726  4 general pract*.mp. 17592  5 United Kingdom.mp. 17942  6 (national health service* or nhs*).mp. [mp=title, abstract, heading word, table of contents, key concepts, original title, tests & measures, mesh word] 8855  7 1 or 2 36  8 3 or 4 17592  9 7 and 8 17  10 limit 9 to yr="2015 -Current" |
| **Web of Science Core Collection (SCIE, SSCI, AHCI)** |
| (All fields) OR general practice OR general practitioner OR national health service OR nhs OR primary care INCLUDE receptionist.  Refined by : Publication Years : 2015 or 2016 or 2017 or 2018 or 2019 or 2020 or 2021 or 2021 or 2022 or 2023 or 2024 or 2025  Languages : English  Countries/Regions : ENGLAND or SCOTLAND or WALES or IRELAND |
| **CINAHL** |
| # Query Limiters/Expanders Last Run Via Results  S10 medical receptionists OR receptionist AND ( general practice or gp or primary care or primary healthcare or primary health care ) AND ( united kingdom or uk or england or britain or scotland or northern ireland or wales ) Limiters - Publication Date: 20150101-20251231  Expanders - Apply equivalent subjects  Search modes - Proximity Interface - EBSCOhost Research Databases  Search Screen - Advanced Search  Database - CINAHL 39  S9 medical receptionists OR receptionist AND ( general practice or gp or primary care or primary healthcare or primary health care ) AND ( united kingdom or uk or england or britain or scotland or northern ireland or wales ) Expanders - Apply equivalent subjects  Search modes - Proximity Interface - EBSCOhost Research Databases  Search Screen - Advanced Search  Database - CINAHL 98  S8 medical receptionists Expanders - Apply equivalent subjects  Search modes - Proximity Interface - EBSCOhost Research Databases  Search Screen - Advanced Search  Database - CINAHL 31  S7 united kingdom or uk or britain or scotland or england or wales or northern ireland Expanders - Apply equivalent subjects  Search modes - Proximity Interface - EBSCOhost Research Databases  Search Screen - Advanced Search  Database - CINAHL 407,166  S6 ( exp General Practice/ or General Practitioners/ or Physicians, Family/ ) OR ( general pract*.ti,ab,kf,jw. or bjgp.jw. ) Expanders - Apply equivalent subjects  Search modes - Proximity Interface - EBSCOhost Research Databases  Search Screen - Advanced Search  Database - CINAHL 81,079  S5 exp General Practice/ or General Practitioners/ or Physicians, Family/ Expanders - Apply equivalent subjects  Search modes - Proximity Interface - EBSCOhost Research Databases  Search Screen - Advanced Search  Database - CINAHL 81,079  S4 ( (receptionist? or reception staff*).mp. ) OR Medical Receptionists Expanders - Apply equivalent subjects  Search modes - Proximity Interface - EBSCOhost Research Databases  Search Screen - Advanced Search  Database - CINAHL 31  S3 (receptionist? or reception staff*).mp. Expanders - Apply equivalent subjects  Search modes - SmartText Searching Interface - EBSCOhost Research Databases  Search Screen - Advanced Search  Database - CINAHL 45  S2 (receptionist? or reception staff*).mp. Expanders - Apply equivalent subjects  Search modes - Proximity Interface - EBSCOhost Research Databases  Search Screen - Advanced Search  Database - CINAHL 0  S1 Medical Receptionists Expanders - Apply equivalent subjects  Search modes - Proximity Interface - EBSCOhost Research Databases  Search Screen - Advanced Search  Database - CINAHL |
| **HMIC Health Management Information Consortium** |
| 1     general practice receptionists/     18  2     exp receptionists/      71  3     (receptionist? or reception staff).mp.    283  4     2 or 3      283  5     exp general practice/   9726  6     exp general practitioners/ or general practice staff/ 10598  7     general pract*.mp.      27441  8     (general practi* or bjgp).jx. 3814  9     5 or 6 or 7 or 8  28260  10    4 and 9     214  11    1 or 10     214  12    limit 11 to yr="2015 -Current" |
| **Overton** |
| “general practice and receptionists” 2015 + , publications only |
