# Supplementary material for: Understanding the contextual and causal factors shaping the work of receptionists in general practice: a realist review protocol
Source: BMJ Open. 2025 Dec 23;15(12):e110991. doi: 10.1136/bmjopen-2025-110991 (PMC12730864; doi:10.1136/bmjopen-2025-110991)
Supplement: Supplementary file 3 [file bmjopen-2025-110991.reviewer_comments.pdf]

PEER REVIEW HISTORY

BMJ Open publishes all reviews undertaken for accepted manuscripts. Reviewers are asked to complete a checklist review form and are provided with free text boxes to elaborate on their assessment. These free text comments are reproduced below.

ARTICLE DETAILS

Title (Provisional)

Understanding the contextual and causal factors shaping the work of receptionists in general practice: A realist review protocol

Authors

Hoverd, Eleanor; Brown, Megan E L; Burford, Bryan; Chen, Ko-Le; Erfani, Goran; Hassan, Sameena; Montgomery, Anthony; Lievesley, Matthew; Norton, John; Owen-Boukra, Emily C; Rapley, Tim; Roberts, Nia; Sajid, Madiha; Sowden, Sarah; Steven, Alison; Vance, Gillian; Park , Sophie

---

VERSION 1 - REVIEW

**Reviewer**	**1**
**Name**	**Beilby, Justin**
**Affiliation**	**National Health and Medical Research Council of Australia Centre of Research Excellence Frailty Trans-disciplinary Research To Achieve Healthy Ageing, Torrens University**
**Date**	**08-Oct-2025**
**COI**	**None**

This is a complex study protocol focussing on an increasingly important team member of the general practice/primary care team - receptionists. I have a number of questions that the authors could consider. I will respond under specific sections.

Abstract

This material does not mention in enough detail that the main focus on this study is on under-served areas.

Introduction

This is well written. I have one comment and some confusion. This relates to table 1 and comments related to this table. The study is focussed on receptionists but Table 1 is headed "Types of non-clinical roles in general practice". The change in language needs more explanation and discussion. I am also confused that the authors then talk about "GP assistants" and "clinical coders" . Are these real receptionists roles ? They maybe in the

changing GP/primary care but there needs to be some discussion in the text underpinning these perceived widening roles.

Methodology

The planned realist review is well described and includes CMOCs, MRTs and in this study protocol is linked to previous work in developing GP workforce sustainability program theory (PT). I have two questions for the authors. Can they please justify why a GP focussed PT will be appropriate for a planned receptionist PT end point.? And is there any other evidence that realists reviews in other health areas have produced valuable research findings that have led to sustained system change. ?

Study Design

All well written and I particularly like the stakeholder, patient and public involvement. The Pawson steps are well summarised in accordance with RAMSES. I have one significant comment re the planned literature search strategy. The authors will mainly focus on UK based literature. They "will consider integrating relevant international literature" - I find this short sighted and I believe will miss learnings from other health systems going through similar primary care change. Europe, Canada and Australia are all going through similar primary care/GP reform with similar general practice frameworks.

VERSION 1 - AUTHOR RESPONSE

<table>
<tr><th colspan="2">Reviewer 1 Prof. Justin Beilby, National Health and Medical Research Council of Australia Centre of Research Excellence Frailty Trans-disciplinary Research To Achieve Healthy Ageing, Torrens University Australia</th></tr>
<tr><td>
Abstract
This material does not mention in enough detail that the main focus on this study is on under-served areas.</td><td>Thank you for identifying that this does not come through strongly enough. We have revised the abstract to read as follows in Lines 39- 50 p2:
ABSTRACT
Background
The work of receptionists in general practice is evolving rapidly and becoming more complex due to a number of changes within primary and community care services, such as increased digitalisation. In under-served areas, these changes have been further complicated by under-resourcing and workforce challenges around staff recruitment and retention. The NHS 10-year health plan is set to accelerate further significant changes. There is limited understanding about how and why these changes and workforce challenges are impacting and will impact the future work of receptionists in general practice in under-served areas.

Methods and analysis This realist review will build upon an existing programme theory related to general practice (GP) workforce sustainability. The review will examine what works, for whom, how and under what circumstances for receptionist work in general practice, in under-served areas. For example, how influences such as the expectations of patients (in under-served communities), poor staffing or limited career progression. Key stakeholders, including public contributors and individuals from general practice settings will inform the realist review.</td></tr>
<tr><td>Introduction
This is well written. I have one comment and some confusion. This relates to table 1 and comments related to this table. The study is focussed on receptionists but Table 1 is headed "Types of non-clinical roles in general practice". The change in</td><td>Thank you for your comments in this section. As this manuscript outlines the study's protocol, the decision to use the broader terminology and to highlight these emerging roles will be fully discussed and underpinned by evidence in the Discussion section of the final findings paper. This discussion will explicitly address the</td></tr>
</table>

language needs more explanation and discussion. I am also confused that the authors then talk about "GP assistants" and "clinical coders" . Are these real receptionists roles ? They maybe in the changing GP/primary care but there needs to be some discussion in the text underpinning these perceived widening roles.	blurring of role boundaries and the expanding scope of administrative work in contemporary general practice.<br>However, to create more clarity in the manuscript we have added the following to **Lines 177-185 p 6**:<br>Widening of the general practice receptionist role, scope of work and the language used to describe a range of roles demonstrates the spectrum of non-clinical support roles that are expanding to cope with the increased pressures on general practice and digitalisation .Table 1 shows the multi-faceted nature of non-clinical support roles, categorising the type of role, title, core functions, and whether interaction with patients is indirect or direct. The work of receptionists may involve a range of these responsibilities with the role of general practice assistants often being filled by experienced receptionists.
Methodology<br>The planned realist review is well described and includes CMOCs, MRTs and in this study protocol is linked to previous work in developing GP workforce sustainability program theory (PT). I have two questions for the authors.<br>- Can they please justify why a GP focussed PT will be appropriate for a planned receptionist PT end point.?	Thank you for raising these important questions. Our review whilst ultimately aims for a receptionist programme theory, is intentionally linked to the existing GP workforce sustainability programme theory as there is a shared organisational and sustainability context and it provides an initial framework to build upon avoiding disconnect from previous knowledge. We have added in further justification that hopefully helps to make this clearer in the manuscript in **336-348 p12-13**<br><br>Building on a previous review and programme theory through taking this approach will strengthen the relevance and generalisability of findings. Whilst aiming to develop a programme theory around the work of receptionists in general practice, the GP workforce sustainability programme theory provides a rigorous, empirically-grounded starting point to explore whether mechanisms are relevant for receptionists and whether context needs to be redefined for the work of receptionist roles. We will also explore how resulting outcomes differ. This will be supported through the realist approach by making inferences through induction, deduction and

	abduction/retroduction, allowing us to identify where there are similarities, differences or extensions of thinking to develop our theories [43]. A co-produced realist review provides a broader foundation for deeper insights and more adaptable implementation strategies, through engagement with a range of knowledge(e.g. experiential knowledge, grey literature and empirical research). This cumulative process enables us to build on insights gained from analysing and synthesising a number of studies and the findings of the GP workforce sustainability review. By identifying new ways of understanding and interpreting these findings, we can apply them meaningfully to shape our future 'Workforce Voices' co-design work across the wider workforce sustainability programme workstreams. Formal theories of change and implementation will provide conceptual scaffolding to guide the realist reviews, co-design and implementation work [43-44].
And is there any other evidence that realists reviews in other health areas have produced valuable research findings that have led to sustained system change. ?	Thank you for also raising this point, it stimulated a great discussion within our research team and is recognised as a gap in the evidence base. As Pawson describes in alignment with Weiss's (1979) 'enlightenment model' of research utilisation : " it is the concepts and theoretical perspectives of social science that permeate policy process...shaping the way people think about social issues."<br><br>The main purpose of our review is as mentioned in **Lines 362-363 p13** :<br><br>"Insights from this review will inform the broader programme logic across the workforce partnership."<br><br>However, to expand further we have added in **Lines 282-288 p10**:<br><br>The review will produce causal explanations as described by Pawson in the form of context-mechanism-outcome configurations, that create a "medium of policy learning" which is under-reported in the literature [36]. This review will also create a practical blueprint for

	the implementation of interventions. This will be applied to the 'Workforce Voices' programme of work and reported on in future publications as well as any findings that have led to system change.
Study Design<br>All well written and I particularly like the stakeholder, patient and public involvement. The Pawson steps are well summarised in accordance with RAMSES. I have one significant comment re the planned literature search strategy. The authors will mainly focus on UK based literature. They "will consider integrating relevant international literature" - I find this short sighted and I believe will miss learnings from other health systems going through similar primary care change. Europe, Canada and Australia are all going through similar primary care/GP reform with similar general practice frameworks.<br>applicable	Thank you for this key point. We agree wholeheartedly that valuable insights could be gained from comparable health systems, particularly those in Europe, Canada, and Australia. However, as the realist review is theory-driven and purposive it does not aim to be exhaustive and so we are taking a focussed , iterative approach to the search.

VERSION 2 - REVIEW

Reviewer 1

Name Beilby, Justin

Affiliation National Health and Medical Research Council of Australia Centre of Research Excellence Frailty Trans-disciplinary Research To Achieve Healthy Ageing, Torrens University

Date 16-Nov-2025

COI

Thank you for opportunity to re-review this paper. I am happy that the authors have responded appropriately to my initial questions. I have no further questions.
